# Genomic Insights into the Microbial Agent *Streptomyces albidoflavus* MGMM6 for Various Biotechnology Applications

**DOI:** 10.3390/microorganisms11122872

**Published:** 2023-11-27

**Authors:** Roderic Gilles Claret Diabankana, Mikhail Frolov, Saparmyradov Keremli, Shamil Zavdatovich Validov, Daniel Mawuena Afordoanyi

**Affiliations:** 1 Laboratory of Molecular Genetics and Microbiology Methods, Kazan Scientific Center of the Russian Academy of Sciences, 420111 Kazan, Russia; m.frolov@knc.ru (M.F.); xkesha93x@mail.ru (S.K.); sh.validov@knc.ru (S.Z.V.); d.afordoanyi@knc.ru (D.M.A.); 2 Tatar Scientific Research Institute of Agricultural Chemistry and Soil Science, FRC Kazan Scientific Center, Russian Academy of Sciences, 420111 Kazan, Russia

**Keywords:** antimicrobial activity, bioremediation, biodegradation, biocontrol, biotechnological applications, *Fusarium oxysporum*, heavy metal, *Streptomyces albidoflavus*

## Abstract

Microbial biotechnology plays a crucial role in improving industrial processes, particularly in the production of compounds with diverse applications. In this study, we used bioinformatic approaches to analyze the genomic architecture of *Streptomyces albidoflavus* MGMM6 and identify genes involved in various metabolic pathways that have significant biotechnological potential. Genome mining revealed that MGMM6 consists of a linear chromosome of 6,932,303 bp, with a high G+C content of 73.5%, lacking any plasmid contigs. Among the annotated genes, several are predicted to encode enzymes such as dye peroxidase, aromatic ring-opening dioxygenase, multicopper oxidase, cytochrome P450 monooxygenase, and aromatic ring hydroxylating dioxygenases which are responsible for the biodegradation of numerous endogenous and xenobiotic pollutants. In addition, we identified genes associated with heavy metal resistance, such as arsenic, cadmium, mercury, chromium, tellurium, antimony, and bismuth, suggesting the potential of MGMM6 for environmental remediation purposes. The analysis of secondary metabolites revealed the presence of multiple biosynthesis gene clusters responsible for producing compounds with potent antimicrobial and metal-chelating activities. Furthermore, laboratory tests conducted under controlled conditions demonstrated the effectiveness of MGMM6 in inhibiting phytopathogenic microbes, decolorizing and degrading aromatic triphenylmethane dyes, particularly Blue Brilliant G250, from wastewater by up to 98 ± 0.15%. Overall, the results of our study highlight the promising biotechnological potential of *S. albidoflavus* MGMM6.

## 1. Introduction

The negative impacts of industrial activities and urbanization, including the escalation of antibiotic resistance, soil degradation, industrial dye and heavy metal pollution, and contamination, are emerging as pressing global issues [1,2,3]. The pollution resulting from these factors is persistent, toxic, and poses a significant threat to the health of living organisms [4,5]. Antimicrobial resistance has emerged as one of the foremost concerns for human health, food safety, and sustainable development. For example, cadmium toxicity in crop plants delays the absorption and movement of nutrients and water, increases the occurrence of oxidative harm, disturbs plant metabolism, and hampers plant structure and function [6]. To address these challenges, researchers around the world are focusing on eco-friendly and economical solutions that can effectively solve this multifaceted problem. Heavy metals, soil remediation, and the discovery of novel sources of new antimicrobial compounds with high antibiotic activity can be managed with an eco-friendly approach using microorganisms [7,8]. Various microbial taxonomic genera, such as *Actinomyces*, *Bacillus*, *Arthrobacter*, and *Photorhabdus,* can survive harsh conditions [9]. They have developed resistance mechanisms such as adsorption of metals on the cell surface, increased activity of efflux pumps, production of extracellular chelating agents (siderophores), intracellular sequestration and biomineralization, and processes such as reduction, oxidation, or methylation that allow them to transform toxic metals into less harmful and more mobile forms [10,11,12,13]. Moreover, during their life cycle, they produce a variety of potentially bioactive compounds with agricultural, pharmaceutical, and biotechnological properties [14,15].

*Streptomyces,* as the largest bacterial genus, is considered a renowned genus for its ability to produce active metabolites (almost 80% of the world’s antibiotics), which makes it a subject of great interest in the development of novel therapeutic, agricultural, pharmaceutical, and biotechnological agents [16]. Several species of this genera isolated from diverse environmental samples are expected to act as plant growth-promoting and control agents against pathogens, as well as agents for biodegradation and bioremediation of insoluble polymers such as lignin and synthetic insecticides [16,17,18]. *Streptomyces albidoflavus* is a Gram-positive filamentous versatile soil bacterium with the ability to form a symbiotic relationship with both plants and animals. Strains of these species are considered remarkable producers of attractive metabolites with antimicrobial and plant growth-promoting activities [19,20,21,22]. Genomic analysis appears to be a promising approach for enhancing the selection of effective bioremediation agents for heavy metal pollutant elimination from environments and the identification of new active compounds with potent antimicrobial activity. In this study, we performed whole-genome sequencing of *S. albidoflavus* MGMM6 and employed bioinformatics approaches to identify genes, gene clusters, and metabolic pathways associated with *S. albidoflavus* MGMM6 which has potential applications in various fields of industrial and environmental biotechnology such as plant protection, biological remediation of dyes.

## 2. Materials and Methods

All microbial strains used in this study were provided by the Laboratory of Molecular Genetics and Microbiology Methods (FRC Kazan Scientific Center, Kazan, Russia). Bacterial strain MGMM6 was isolated from the rhizosphere soil of spring wheat (*Triticum aestivum* L.) Yoldyz variety (Republic of Tatarstan, Kazan, Russia). For this purpose, a 100 µL 5-fold-dilution of rhizospheric soil (100 mg) was performed with phosphate-buffered saline [(PBS): 140 mM NaCl, 5 mM KH_2_PO_4_, 1 mM NaHCO_3_, pH 7.4], plated on Gause #2 medium [(g/L): 2.5 g tryptone, 5 g peptone, 5 g NaCl, 10 g glucose, 20 g agar, pH 7.4], and incubated at 30 °C for 72 h. After incubation, single-growth colonies were replated on Gause #2 medium for molecular identification using 16S rRNA. Among the isolated bacterial strains, MGMM6 was identified as *Streptomyces albidoflavus* (GenBank: OR822207) and was further used in this study for its various biotechnology applications.

### 2.1. Phenotypic Characterization

Phenotypic characterization of MGMM6 was evaluated based on colony morphology (shape, color, and other characteristics such as form and transparency) after growth on KB (20 g Proteose peptone; 1.5 g K_2_HPO_4_; 0.75 g MgSO_4_ × 7H_2_O; 10 mL glycerol; 20 g agar, pH 7.2 ± 0.2 at 25 °C) and (International Streptomyces Project medium (ISP)-4 [(g/L): 10 g soluble starch; 1 g MgSO_4_ 7H_2_O; 1 g NaCl; 2 g (NH4)_2_SO_4_; 2 g CaCO_3_. 1 mL trace salt solution (0.1 g FeSO_4_ × 7H_2_O; 0.1 g MnCl_2_ × 4H_2_O; 0.1 g ZnSO_4_ × 7H_2_O; 100 mL dH_2_O; 20 g agar)] medium. For this purpose, an overnight culture of MGMM6 grown on Gause #1 [(g/L): 20 g soluble starch; 1 g KNO_3_; 0.5 g NaCl; 0.5 g K_2_HPO_4_; 0.5 g MgSO_4_; 0.01 g FeSO_4_; 20 g agar] was streaked on KB and ISP-4 agar medium and incubated for up to 10 days at 30 ± 1 °C. After incubation, colony morphology was analyzed. All experiments were performed in triplicate.

### 2.2. Library Construction, Genome Sequencing, and Analysis

The genomic DNA of *S. albidoflavus* MGMM6 was isolated using a phenol-chloroform extraction method [23] and cleaned using a cleanup kit (Evrogen, Moscow, Russian Federation), according to the manufacturer’s instructions. The whole genome was sequenced using an Illumina HiSeq 2500 System with 2 × 125 bp paired-end reads. Low-quality reads, including adapters and N > 5% reads, were removed using Trimmomatic v. 0.36 and FastP v. 0.23.4-2 [24,25]. The genome was assembled using RAPT (Read Assembly and Annotation Pipeline Tool), Unicycler v.0.3.0, and SPAdes v. 3.15.4 [26,27,28]. Tetra correlation search using the web server tool JSpeciesWS (https://jspecies.ribohost.com/jspeciesws/#home, accessed on 20 March 2023) based on ANIb (average nucleotide identity based on BLAST) was used to select the close-related reference strain. A web server for contig scaffolding using algebraic rearrangements [29] was used to reorder contigs based on a comparison with the reference genome. The gaps in the scaffolds were filled and closed using GapBlaster and ntedit_sealer [30]. The presence of plasmids in the sequenced genome of MGMM6 was analyzed using Plasmid Spades [31], whereby Mob-recon [32,33] was applied to identify the presence of plasmids in the pre-assembled genomic contigs generated by Unicycler and SPAdes v. 3.15.4. Automated annotation of *S. albidoflavus* MGMM6 and the open reading frame was performed using Bakta v.1.8.1 and the NCBI Prokaryotic Genome Annotation Pipeline (PGAP) [34,35]. Quality assessment of *S. albidoflavus* MGMM6 was performed using CheckM v1.2.2 [36]. The detection of antimicrobial resistance genes in *S. albidoflavus* MGMM6 was predicted using the Comprehensive Antibiotic Resistance Database (CARD) [37]. The functional and organization of gene clusters involved in the synthesis of secondary metabolites (BGC) were analyzed using AntiSMASH v. 7.0 [38].

The xenobiotic biodegradation and biosynthesis pathways of prospective compounds with plant-beneficial functions involved in MGMM6 were analyzed using RAST and the KEGG Orthology-based Annotation System. MobileOG-db (beatrix-1.6) with default parameters [39] was used to analyze the genome of *S. albidoflavus* MGMM6 for the presence of mobile orthologous genes mediating integration/deletion, replication/recombination/repair, stability/protection, or transfer of bacterial mobile genetic elements and phages, as well as transcription regulators related to these processes.

### 2.3. In Silico Identification of Heavy Metal Resistance Genes

The identification of heavy metal and biocide resistance genes in MGMM6 was conducted using AMRFinderPlus v. 3.10 and BacMet [40,41]. Since heavy metal operons and AMR genes can often be found within bacterial chromosomes or plasmids, the annotated assembly of MGMM6 was used. The operons arsRBC were used as a reference to predict the presence of arsenic resistance in MGMM6. The operon PcoABCDSRE (multicopper oxidase PcoA, copper binding protein PcoB, copper resistance system metallochaperone PcoC and E, copper response regulator transcription factor PcoD, and copper resistance membrane protein PcoS) was used for copper. Operon cmtR (metal-responsive transcriptional repressor for the cmt operon) and cnrA (Fragment of nccA-like protein) were used as a reference to predict the ability of MGMM6 to be resistant to Co and Ni. The presence of metal genes resistant to antimony (Sb)-arsAB, bismuth-arsR, and cadC; cadmium (Cd)-actSR; chromium-chrA; cobalt-cmeAB; copper-actARP and baeR; gallium (Ga)-fbpABC; gold (Au)-gesA; lead (Pb)-cadC and nmtR; mercury (Hg)-dsbAB and merA; molybdenum (Mo)-modAB; selenium (Se)-recG, ruvB, and sodA; silver (Ag)-copAB, cueA, or others; tellurium (Te)-actP, tungsten (W)-baeRS, and modAB; vanadium (V)-mexI, perO, and yieF were analyzed.

### 2.4. Antibiosis Activity

The antibiosis assay of *S. albidoflavus* MGMM6 against the pathogens *Fusarium oxysporum* f. sp. *radicis-lycopersic* (*Forl*) ZUM2407, and *F. proliferatum* was performed using an agar diffusion assay on King’s B medium according to Bonev et al. [42]. *Pseudomonas putida* PCL1760 and *Bacillus velezensis* KS04AU were used as negative and positive controls, respectively.

### 2.5. Ability of S. albidoflavus MGMM6 to Decolorize and Degrade Dyes

**Figure 1:**
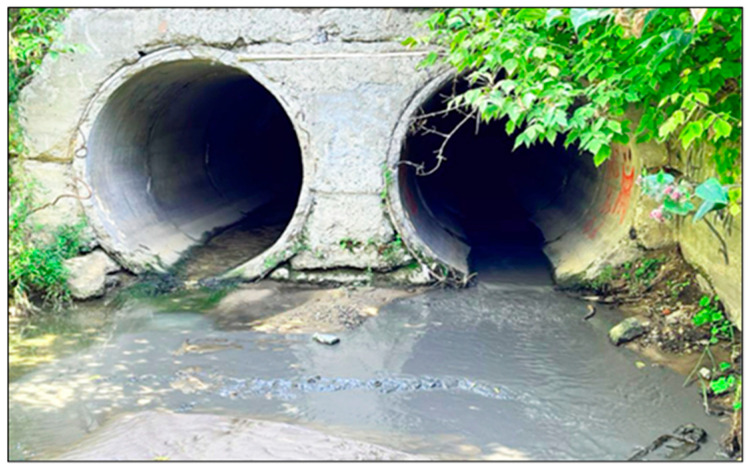
Wastewater used in this study to mimic the natural environment to evaluate the dye decoloration and degradation ability of *S. albidoflavus* MGMM6.

The dye removal ability of MGMM6 was quantitatively (based on visual dye-discoloration) and qualitatively (by measuring the percentage of degraded dye) evaluated according to Aravind et al. [43]. For this purpose, an overnight culture of MGMM6 (with a cell density of 0.5 at 595 nm) was inoculated with a ratio of 1/100 in a two-fold diluted nutrient broth (NB) [(g/L) meat extract 1.0; peptone 5.0 g; NaCl 5.0 g; yeast extract 2.0 g; pH 7.4)] and wastewater medium (pH 8.0) amended with 1% (*v*/*v*) of Coomassie Brilliant Blue G-250 solution (prepared by dissolving 1 g of Coomassie Brilliant Blue G-250 in water and sterilized by filtration (passed through a 0.22 μm membrane filter (Type Millex-HA, Millipore, Burlington, MA, USA). Wastewater medium was prepared from wastewater sample (Figure 1) left at room temperature for one day for soil precipitation. After precipitation, the excess water was collected and used as a medium. Non-inoculated media were used as controls. All experiments were performed in triplicate.

The obtained solution was incubated at 28 ± 1 °C for 7–14 days. After incubation, the cultures were centrifuged at 10,000 rpm for 10 min at room temperature. The obtained supernatants were used to determine the dye degradation percentage. The degradation of dye (dye removal) was measured using a UV/VIS spectrophotometer nanodrop with wavelength intervals from 534 to 716 nm. Non-inoculated media were used as blanks. The dye removal percentage (%) was expressed using the following formula:

$$Dye\;removal\left(\%\right)=\frac{Initial\;absorbance-Final\;absorbance}{Initial\;Absorbance}\times 100$$

### 2.6. Statistical Analysis

Statistical analysis of these obtained data was performed using the statistical program OriginLab pro-SR1 b9.5.1.195 (OriginLab Corp., Northampton, MA, USA). To determine the significant difference between groups, a one-way ANOVA and post hoc Tukey’s honestly significant difference test (*p* < 0.05) were conducted.

## 3. Results

### 3.1. Phenotypic Characterization and Genome Sequencing Analysis 

**Figure 2:**
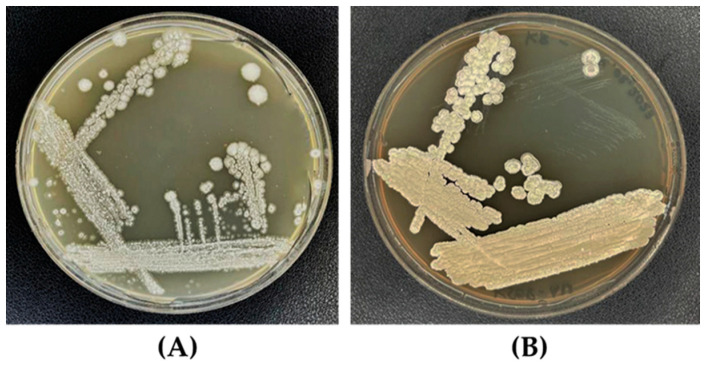
Colony morphology of *S. albidoflavus* MGMM6 on KB (**A**) and ISP-4 (**B**) agar medium. Plates were incubated for up to 10 days at 30 ± 1 °C.

After incubation for 10 days at 30 ± 1 °C, the colony of *S. albidoflavus* MGMM6 developed buried colonies with hard consistency and dry appearance (Figure 2) and white (on ISP-4 agar medium, Figure 2A) and slightly whitish–yellow color (on KB agar medium, Figure 2B).

The genomic assembly of *S. albidoflavus* MGMM6 comprised a linear chromosome of 6,932,303 bp, with a high G + C content of 73.5% without plasmid contigs. Tetra correlation search using the Web server tool JSpeciesWS showed that *S. albidoflavus* MGMM6 was closely related to *S. albidoflavus* DSM 40233 (NCBI RefSeq assembly: GCF_004195775.1; BioSample ID: SAMN08225702) with 98.96% similarity based on ANIb.

**Figure 3:**
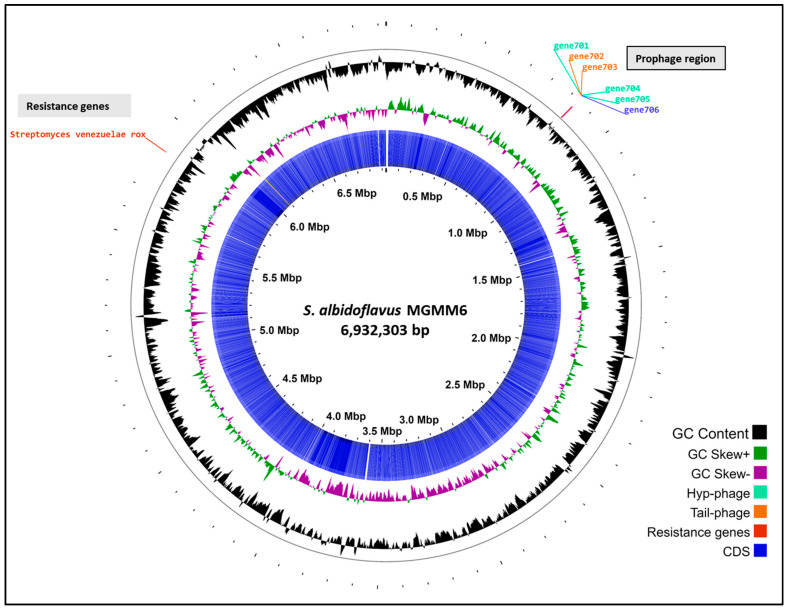
The in silico circularization of the linear chromosome of *S. albidoflavus* MGMM6.

Automated annotation using PGAP revealed that the genome of MGMM6 carries a total of 6024 genes, with 5851 coding genes. *Streptomyces albidoflavus* MGMM6 contains 69 genes (RNA), 63 of which are transfer ribonucleic acid (tRNA) and three are non-coding RNA (ncRNA)]) (Figure 3). The presence of 104 pseudogenes in *S. albidoflavus* MGMM6 was predicted using PGAP. Automated annotation using Bakta predicted the presence of 5949 coding genes, 64 genes (RNA), 11 pseudogenes, and 2 CRISPR arrays. The origin of replication (*oriC*) was predicted to be in the genome region from 5,331,504 to 5,332,639. Qualitative analysis using CheckM revealed that the completeness of the genome was 99.34% (Table 1). 

Protein-encoding genes involved in chitin degradation, oxidation cleavage of glycosidic bonds, and reversible phosphorolysis of α-1,4-linked polysaccharides, namely chitinase, lytic polysaccharide monooxygenase, cellulase family glycosyl-hydrolase, and alpha-glucan family phosphorylase, were annotated in MGMM6. Moreover, a high proportion of genes involved in the degradation of aromatic compounds, such as multicopper oxidase, cytochrome P450 monooxygenase, DyP-type peroxidase, aromatic ring hydroxylating dioxygenases, aromatic ring-opening dioxygenase LigA, and nitric oxide dioxygenase, which play a key role in the biodegradation of numerous environmental pollutants, were predicted in *S. albidoflavus* MGMM6. The gene encoding 1-aminocyclopropane-1-carboxylate deaminase enzyme, which plays an important role in reducing biotic and abiotic stress in plants, was present in the annotated genome of *S. albidoflavus* MGMM6. The nitronate monooxygenase gene, a flavin-dependent enzyme that catalyzes the denitrification of propionate 3-nitronate (P3N) and other alkyl nitronates, was identified in the genome of MGMM6.

**Figure 4:**
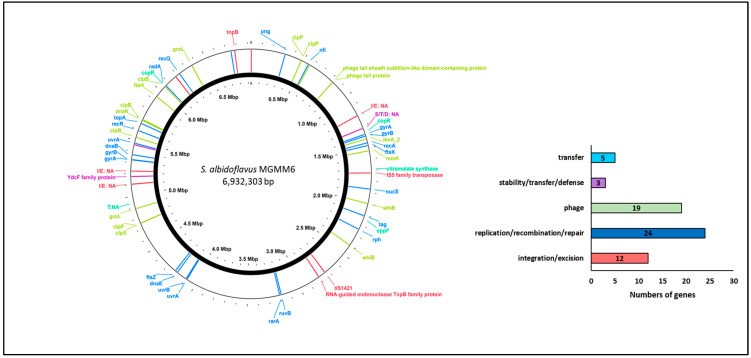
Identification of genes associated with integration/excision, replication/recombination/repair, stability/defense, or transfer of bacterial genetic elements and phages.

The analysis of gene families associated with integration and excision, replication and recombination, DNA repair, stability, defense, and the transfer of bacterial genetic elements and phages is presented in Figure 4. The results revealed that *S. albidoflavus* MGMM6 harbors 24 genes involved in the replication, recombination, or repair of mobile genetic elements, such as plasmid replication. Five genes associated with the mediation of inter-organism transfer of bacterial genetic elements were found in the genome of MGMM6. The presence of 12 genes (associated with integration and deletion) that control, mediate, or assist in site-specific recombination of genetic elements was predicted in *S. albidoflavus* MGMM6. The presence of 19 genes associated with the biological processes of bacteriophages, such as viral genome packaging lysis and lysogeny-associated machinery, was detected in MGMM6.

The xenobiotic biodegradation pathway involved in MGMM6 is presented in Table 2. The obtained result revealed that *S. albidoflavus* MGMM6 harbors genes involved in the degradation of 1,3,4,6-Tetrachloro-1,4-cyclohexadiene to 2,5-Dichloro-2,5-cyclohexadiene-1,4-diol via alpha/beta hydrolase (LinB). In addition, among the distinct enzyme commission (EC) numbers involved in the metabolism of xenobiotics by cytochrome P450, 42.9% of the degradation pathway was predicted in the genome of MGMM6. Two (28.6%) ECs were associated with trinitrotoluene degradation. In addition, we found that MGMM6 can degrade (1R,2S)-Naphthalene-1.2-oxide to (91R)-OH-(2R)-Glutathionyl-1,2-dihydronaphthalene and (1R,2S)-Naphthalene-1.2-oxide to 1,2-Dhydroxy-1,2-dihydronaphthalene via epoxide hydrolase (EC 3.3.2.9), glutathione S-transferase (EC 2.5.1.18), alcohol dehydrogenase (EC 1.1.1.1), and acetaldehyde dehydrogenase (EC 1.2.1.10). The degradation ability of 1,2 dibromoethane and 2-bromo-acetaldehyde to S-[2-(N7-Guanyl) ethyl] N-acetyl-L-cysteine and S-(Formylmethyl)-glutathione via glutathione S-transferase (EC 2.5.1.18) was also found in the xenobiotic biodegradation pathway of *S. albidoflavus* MGMM6. The xenobiotic biodegradation of 4-nitrophenyl-phosphate to 4-nitrophenol via 4-nitrophenylphosphatase was predicted (Table 2).

**Figure 5:**
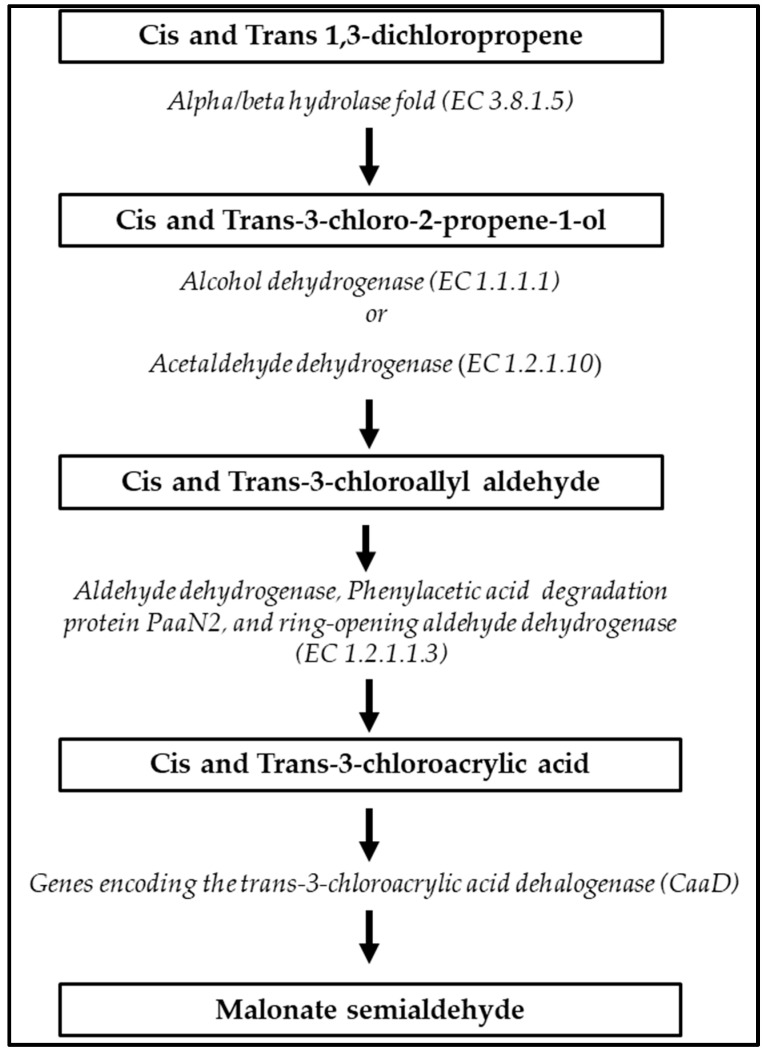
Xenobiotic biodegradation pathway of cis and trans-1,3-dichloropropene by *S. albidoflavus* MGMM6.

A full-degradation pathway of xenobiotic cis and trans-1,3-dichloropene, in which five distinct ECSs are involved, was predicted in *S. albidoflavus* MGMM6 (Figure 5). First, cis and trans-1,3-dichloropene are hydrolyzed into cis and trans-3-chloro-2-propene-1-ol; second, cis and trans-3-chloro-2-propene-1-ol are dehydrogenated to cis and trans-3-chlorophyll aldehyde, and then dehydrogenated to cis and trans-3-chloroacetic acid. Finally, cis and trans-3-chloroacetic acid undergoes dehydration to malonate semialdehyde. 

Biosynthesis pathways of various metabolites with potentially beneficial activity for plants, including plant hormones, were predicted in the genome of *S. albidoflavus* MGMM6 (Table 3). The results showed that among various ECSs, MGMM6 produces 74 (56.5%) ECSs involved in the biosynthesis of plant hormones and 2 (66.7%) in the biosynthesis of brassinosteroids. The presence of pathways involved in the synthesis of auxin, abscisic acid, cytokinins, salicylic acid, gibberellin, strigolactone, jasmonic acid, and ethylene was predicted in MGMM6.

Analysis of clusters involved in the biosynthesis of secondary metabolites using antiSMASH predicted the presence of 21 gene clusters in *S. albidoflavus* MGMM6 (Table 4).

Fifteen regions with similarity scores > 75% matched to the most similar known cluster were predicted in *S. albidoflavus* MGMM6. Among them, biosynthetic gene clusters (BGCs) encoding NRPS, terpene, ectoine, lanthipeptide-class-II and III, and NI-siderophore were found, which are responsible for the synthesis of antimycin, candicidin, minimycin, isorenieratene, ectoine, surugamide A/D, fredericamycin A, ectoine, hopene, AmfS, geosmin, and cyclofaulknamycin. Five BGCs encoding fNRPS, terpene/NRPS/NRPS-like, RiPP-like, and thiopeptide/LAP/RRE-containing peptides with less similarity (<70%) to most known BGCs were predicted to produce dudomycin A, fluostatins M-Q, hexacosalactone A, synechobactin, and julichrome.

### 3.2. Identification of Antimicrobial and Heavy Metal Resistance Genes in S. albidoflavus MGMM6

We analyzed the presence of heavy metal resistance and antimicrobial resistance genes harboring *S. albidoflavus* MGMM6 using bioinformatic approaches. Resistome analysis of *S. albidoflavus* MGMM6 using the CARD system revealed the presence of rifampin monooxygenase AMR gene family, which provides resistance to rifamycin antibiotic groups. This resistance occurs through the mechanism of antibiotic inactivation.

Several genes related to heavy metals were identified in the MGMM6 genome (Table 4). Genes providing resistance to copper, including multicopper oxidase, heme-copper oxidase subunit II, copper homeostasis cutC, copper ion binding), and copper chaperone PCu(A)C, were predicted in MGMM6. The annotated genome of MGMM6 harbors three arsenate reductase (arsC) genes that reduce arsenate As(V) to arsenite As(III). The operons recG and ruvB, which provide resistance to tellurium and selenium and are involved in repairing DNA damage caused by chromate or its derivatives, were identified in MGMM6. The gene *modA* involved in the transport of molybdenum and tungsten into cells was predicted in MGMM6. Two magnesium and cobalt transport proteins, CorA, which mediate the influx and efflux of magnesium, cobalt, and nickel ions, were identified in MGMM6. The F-box motif-containing protein (FBP) domain-containing protein, DsbA family protein, and 4 DsbA family oxidoreductases genes conferring resistance to cadmium, zinc, and mercury were predicted in *S. albidoflavus* MGMM6. Two magnesium and cobalt transport *CorA* operons that also confer resistance to magnesium, cobalt, nickel, and manganese were identified in MGMM6. In contrast, genes associated with resistance to vanadium and gold were not identified in the MGMM6 genome.

### 3.3. Antibiosis Activity

**Figure 6:**
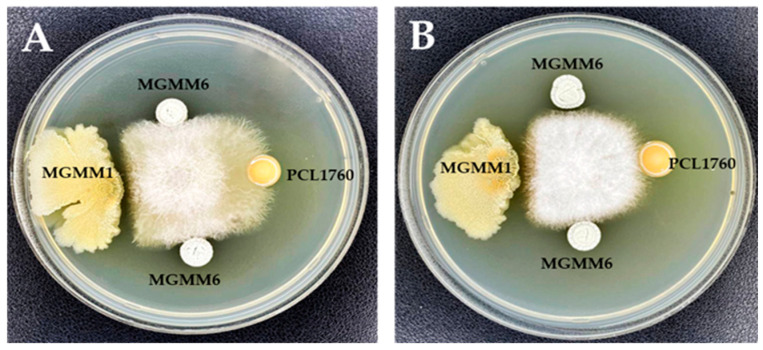
Antimicrobial activity of *S. albidoflavus* MGMM6 against (**A**) *F. oxysporum* f. sp. *radicis-lycopersici* ZUM2407, and (**B**) *F. proliferatum*.

The antimicrobial activity of MGMM6 is shown in Figure 6. The obtained results indicate that MGMM6 inhibits the growth of the tested pathogens. A significant antagonistic effect was observed against the phytopathogen Fusarium oxysporium (Figure 6A).

### 3.4. Dye Decolorization and Degradation Ability

**Figure 7:**
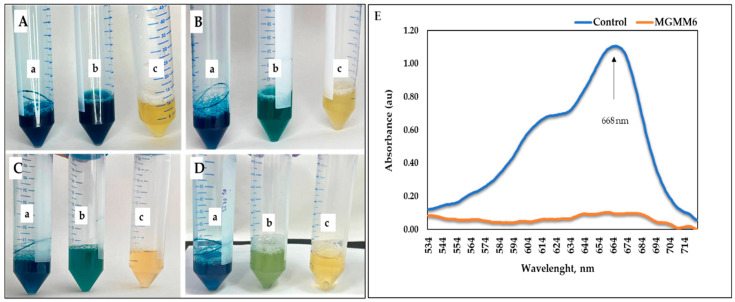
*S. albidoflavus* MGMM6 was inoculated in a medium amended with 1% (*v*/*v*) Coomassie brilliant blue G-250 solution. (**a**) NB + 1% (*v*/*v*) of Coomassie brilliant blue G-250 solution; (**b**) NB + 1% Coomassie brilliant blue G-250 solution + MGMM6; (**c**) NB medium. Dye decolorization after inoculation (**A**) and 2 days (**B**), 4 days (**C**), and 7 days (**D**) after incubation at 28 ± 1 °C. The UV–vis spectra registered on the spectrophotometer 7 days after incubation (**E**)—where control and MGMG6 represent the UV–vis spectra of NB + 1% (*v*/*v*) of Coomassie brilliant blue G-250 solution and NB + 1% Coomassie brilliant blue G-250 solution + MGMM6.

**Figure 8:**
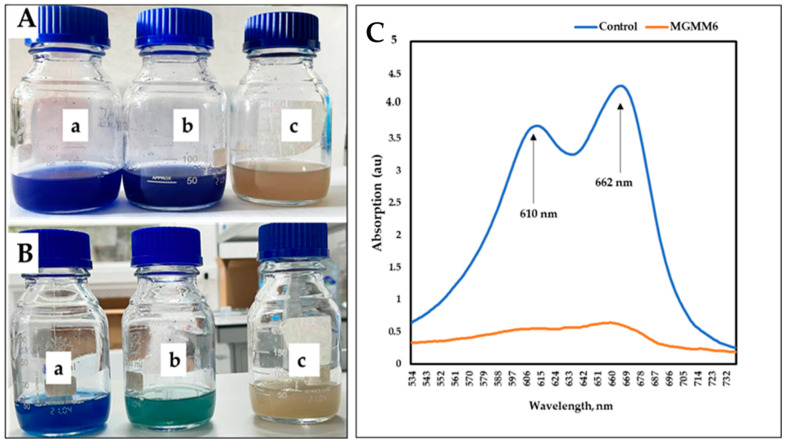
The dye decolorization and degradation ability of *S. albidoflavus* MGMM6. Dye decolorization after one (**A**) and 14 (**B**) days of incubation at 28 ± 1 °C. The absorption spectrum of dye degradation after 14 days of incubation (**C**)—where control and MGMG6 represent the UV–vis spectra of wastewater + 1% (*v*/*v*) of Coomassie brilliant blue G-250 solution and wastewater + 1% Coomassie brilliant blue G-250 solution + MGMM6. *Streptomyces albidoflavus* MGMM6 was inoculated in wastewater amended with 1% (*v*/*v*) Coomassie brilliant blue G-250 solution. Wastewater + 1% (*v*/*v*) Coomassie brilliant blue G-250 solution (**a**); Wastewater + 1% (*v*/*v*) Coomassie brilliant blue G-250 solution + MGMM6 (**b**); wastewater (**c**).

The ability of MGMM6 to decolorize and degrade dye is shown in Figure 7 and Figure 8. The results show that MGMM6 statistically decolorizes and degrades dyes. After incubation, decolorization of the solution was observed without changing the hydrogen index (pH) (Figure 7A–D). In addition, compared with the control, the UV–vis spectra of Coomassie brilliant blue G-250 of groups pre-treated with MGMM6 were not registered. Their absorption values were assayed as 1.51 ± 0.15 and 0.05 ± 0.03 au, respectively (Figure 7E). The dye removal ability of MGMM6 was measured as 96.67% ± 0.46.

Similar results were obtained using wastewater amended with 1% Coomassie Brilliant Blue G-250 (Figure 8A,B). After 14 days of incubation, two distinct peaks were detected in the control group at 610 and 662 nm, with absorption values of 4.28 ± 0.25 and 0.68 ± 0.05 a.u (absorbance units), respectively (Figure 8C). In comparison, the group pretreated with MGMM6 showed a slight UV-Vis spectrum of Coomassie Brilliant Blue G-250 at 662 nm, with a value of 0.63 a.u. The effectiveness of *S. albidoflavus* MGMM6 in degrading and decolorizing wastewater amended with 1% (*v*/*v*) Coomassie Brilliant Blue G-250 was up to 85.98 ± 0.25%. The effectiveness of *S. albidoflavus* MGMM6 in degrading and decolorizing wastewater amended with 1% Coomassie Brilliant Blue G-250 solution was up to 85.98 ± 0.25%.

## 4. Discussion

Microbial biotechnology is an emerging science field with significant potential applications, including food security, human health and nutrition, waste management, and plant protection [44,45,46]. The application of microbial strains in these related fields mainly depends on the specific arrangement of their genetic material, including the presence of certain genes and regulatory elements. In the context of potential sources of compounds with antimicrobial activity, according to the results of this extensive study, these abilities were identified in the genome of MGMM6 (Figure 6 and Table 3). The ability of *S. albidoflavus* MGMM6 to inhibit the growth of pathogens is mainly related to the presence of these putative gene clusters responsible for the synthesis of cyclic peptides such as valinomycin/montanastati, cyclofaulknamycin, hexacosalactone, and antimycin. These compounds possess strong antimicrobial activity against bacterial and fungal pathogens [47,48,49,50,51,52,53]. For example, surugamides and their derivatives, such as acyl-surugamide A, possess anticancer and antifungal activities [53,54]. Desferrioxamine B, which is a cation metal chelator, has been reported to be used in several medicinal and analytical applications, such as aluminum chelation therapy in people on dialysis [55,56]. Hence, *S. albidoflavus* MGMM6 can be applied in agriculture as a biocontrol agent and chelating agent to eliminate the deficiency of metals, such as iron, in plants. Polycyclic tetramine macrolactams (SGR PTM) and antimycin are well-known compounds secreted by streptomyces with fungicidal, insecticidal, and acaricidal activities [57,58]. *Fusarium* is a well-known genus that causes significant agricultural yield losses, reaching up to 14% annually [59,60,61]. Species of this genus produce mycotoxins in food and agricultural products and are suspected to be associated with various diseases in mammals and other organisms [62,63]. In this study, through dual-plate assay, *S. albidoflavus* MGMM6 inhibited the growth of *Fusarium* species. The obtained result consistent with these previously reported by [20], whereby *S. albidoflavus* strain CARA17 isolated from root diseases of grapevine plants (*Vitis vinifera*) showed the ability to inhibit the growth of fungal soil-borne pathogens such as *Athelia rolfsii*, *F. oxysporum*, *Plectosphaerella ramiseptata*, *Sclerotinia sclerotiorum,* and *Verticillium dahlia*. The role of microbial biotechnology in sustainable agriculture and environmental health is well documented. Microbial agents assimilate and acquire plant-essential nutrients, remediate and improve soil physicochemical properties, modulate the synthesis of plant hormones, and produce various signal compounds that inhibit the growth of several pathogens, as well as improve plant activities under stress conditions [64,65,66]. Moreover, through bioinformatics approaches, several beneficial plant genes responsible for degrading xenobiotic compounds, remediating heavy metals, and metabolic pathways involved in plant hormones and lytic enzyme synthesis were predicted in *S. albidoflavus* MGMM6. These genes were found to be involved in several biodegradation processes. Castillo et al. [67] reported the efficient ability of *S. albidoflavus* strain A7-9 to degrade up to 100% 4 mg/L of N′-3,4-Dichlorophenyl-N, N-dimethylurea (an herbicide of the aryl-urea class) for 15 days at 25 °C. These abilities to use aromatics as the sole carbon and energy sources are related to the presence of genes such as cytochrome P450 monooxygenase (P450s), oxidase, lignin peroxidase, manganese peroxidase, DyP-type peroxidase, versatile peroxidase, humic acid peroxidases, dioxygenase, and laccase [68,69,70,71,72]. The above genes were also found in the genome of *S. albidoflavus* MGMM6, which may refer to its ability to decompose the above aromatic compounds, as in the strain A7-9 of *S. albidoflavus* [67].

Considering the presence of these genes in the genome of MGMM6, we were interested in testing its ability to degrade polycyclic aromatic compounds such as triphenylmethane dyes. Different microorganisms isolated from different taxonomic groups have demonstrated several catabolic activities in the degradation of hazardous materials such as 2,4-dinitrophenol, 2,4,6-trichlorophenol, and pyridine [73,74]. In this study, *S. albidoflavus* MGMM6 demonstrated the ability to decolorize and degrade Coomassie Brilliant blue R250 from wastewater at 28 ± 1 °C at a constant pH (8.0). The obtained results correlated with previously reported data. For example, Musengi et al. [75] isolated an extracellular DyP-type peroxidase class from *S. albidoflavus* BSII#1, which showed the ability to degrade and decolonize reactive blue 4, reactive black 5, and Azure, as well as, to oxidize 2,4-dichlorophenol, 2,4-dichlorophenol, 2,6-dimethoxyphenol, 4-tert-butylcatechol, ABTS [2,2′-azino-bis(3-ethylbenzothiazoline-6-sulfonic acid)], caffeic acid, catechol, guaiacol, L-DOPA, o-aminophenol, phenol, and pyrogallol. Similar results were obtained by Alaidaroos et al. [76], whereby *Streptomyces flavus* BA4 isolated from wastewater showed the ability to remove chromium up to 300 mg/L.

The survival and behavior of bacteria in soil mainly depend on their genomic architecture, including their resistome, mobilome, and metabolic features. Numerous studies have shown that antibiotics harm soil microbial communities [39,77,78,79,80]. For example, Liu et al. [81] found that sulfamethazine negatively affects the phosphatase activity and respiration of soil microflora. Shan et al. [82] demonstrated that the tetracycline antibiotic group can affect the denitrification process of soil microbial communities. Moreover, analysis of various genomes has shown that species with a lower number of transposons tend to exhibit greater genomic stability [83,84].

Microbes can be successfully used to sequester heavy metals from environments [85,86]. In this study, through bioinformatic methods, we found that MGMM6 harbors several heavy metal resistance genes, such as cadmium, mercury, chromium, tellurium, arsenic (As), and antimony (Sb). These findings are consistent with those reported by Kaliyaraj et al. [87], whereby *S. albidoflavus* T N10 isolated from insect nests demonstrated the ability to recover various heavy metals, including Ca, Cu, Cd, Ni, Ag, and Pb, under laboratory conditions. Thus, these results suggest that *S. albidoflavus* is a promising candidate for its application as a heavy metal remediator. Understanding the relationship between microbial metabolites and their host plants is crucial for developing effective biological agents [88,89]. *Streptomyces albidoflavus* MGMM6 uses multiple metabolite pathways, such as siderophore secretion, nitrogen fixation, and phytohormones, to establish itself in the rhizosphere, compete with other microorganisms in the soil, and protect the plant from pathogens. Furthermore, the limited number of transposable elements within the genome of MGMM6, along with the presence of genes involved in replication, recombination, and metabolic pathways for the degradation of xenobiotic pollutants such as cis and trans-1,3-chloroprene, provide advantages in the genetic manipulation of MGMM6 for various biotechnology applications to plant protection, bioremediation, and biodegradation of persistent organic pollutants. The versatility of *S. albidoflavus* MGMM6 makes it an excellent model organism for novel biotechnological applications.

## 5. Conclusions

Microbiological biotechnology has numerous applications across various scientific fields, each of which can benefit from the unique capabilities of microorganisms. In agriculture, microbiological biotechnology can lead to the development of more resilient and productive crops through the manipulation of plant-microbe interactions, nutrient cycling, and pest management. In this study, *S. albidoflavus* MGMM6 was demonstrated to be an effective bioagent for degrading xenobiotic compounds, remediating heavy metals, and controlling pathogen growth. In addition, the genome of MGMM6 harbors contains several genes involved in replication, recombination, and metabolic pathways for the degradation of xenobiotic pollutants. These features provide distinct advantages for genetic manipulation, making *S. albidoflavus* an attractive candidate for various biotechnology applications such as plant protection, bioremediation, and engineering manipulation of secondary metabolite biosynthesis.

## Figures and Tables

**Table 1:** Summary of genome assembly and annotation of *S. albidoflavus* MGMM6.

Assembly	
Number of contigs (≥1000 bp)	165
Total length (≥0 bp)	6,955,497
Total length (≥1000 bp)	6,942,569
Number of contigs	176
Largest contig	236,127
Total length	6,950,511
GC (%)	73.38
N50	80,168
N90	20,631
L50	30
L90	96
**Annotation**	
Genome Size	6,932,303 bp
Genes (total)	6024
CDSs (total)	5955
Genes (coding)	5851
CDSs (with protein)	5851
Genes (RNA)	69
tRNAs	63
Pseudo Genes (total)	104
Completeness	99.34%
Contamination	1.01%

**Table 2:** Analysis of xenobiotic biodegradation predicted in the genome of *S. albidoflavus* MGMM6.

KEGG Map	Distinct ECs	*S. albidoflavus* MGMM6
Cis and trans 1,3-dichloropropene	4	4 (100%)
2,4-Dichlorobenzoate degradation	29	2 (6.9%)
Benzoate degradation via hydroxylation	50	10 (20.0%)
Naphthalene and anthracene degradation	22	5 (22.7%)
gamma-Hexachlorocyclohexane degradation	28	5 (17.9%)
Benzoate degradation via CoA ligation	44	15 (34.1%)
Benzoate degradation via hydroxylation	50	10 (20.0%)
Biphenyl degradation	13	2 (15.4%)
Toluene and xylene degradation	23	2 (8.7%)
Ethylbenzene degradation	11	3(27.3%)
1,1,2,2-tetrachloroethane degradation	7	1 (14.3%)
Trinitrotoluene degradation	7	2(28.6%)
Fluorobenzoate degradation	10	1 (10.0%)
1- and 2-Methylnaphthalene degradation	17	7 (41.2%)
Metabolism of xenobiotics by cytochrome P450	7	3 (42.9%)
Glycosaminoglycan degradation	16	4 (25.0%)
Fluorene degradation	13	2 (15.4%)

**Table 3:** Biosynthesis pathways predicted in the genome of *S. albidoflavus* MGMM6.

KEGG Map	Distinct ECs	*S. albidoflavus* MGMM6
Brassinosteroid biosynthesis	3	2 (66.7%)
Biosynthesis of plant hormones	131	74 (56.5%)

**Table 4:** Secondary metabolic gene clusters predicted in the genome of *S. albidoflavus* MGMM6.

Region	Type	Most Similar Known Cluster	Representative Class	Similarity
**1**	terpene, NRPS, NRPS-like	valinomycin/montanastatin	NRP + Saccharide: Hybrid/tailoring saccharide	13%
**2**	T1PKS,NRPS	SGR PTMs/SGR PTM Compound b/SGR PTM Compound c/SGR PTM Compound d	NRP + Polyketide	100%
**3**	terpene	hopene	Terpene	76%
**4**	RiPP-like	hexacosalactone A	Other	4%
**5**	NRPS	cyclofaulknamycin	Polyketide	100%
**6**	NI-siderophore	synechobactin C9/C11/13/14/16/A/B/C	Other	9%
**7**	terpene	geosmin	Terpene	100%
**8**	terpene	Julichrome Q3-3/Q3-5	Polyketide	25%
**9**	thiopeptide, LAP, RRE-containing	fluostatins M-Q	Polyketide	4%
**10**	NRPS-like	minimycin	NRP + Saccharide	80%
**11**	RiPP-like, terpene	isorenieratene	Terpene	75%
**12**	RiPP-like, T3PKS	streptavidin	RiPP: Other	75%
**13**	T1PKS,NRPS-like	candicidin	NRP + Polyketide	95%
**14**	T1PKS,NRPS,lanthipeptide-class-ii,NRPS-like	antimycin	NRP:Cyclic depsipeptide+Polyketide:Modultype I polyketide	100%
**15**	ectoine	ectoine	Other	100%
**16**	lanthipeptide-class-iii	AmfS	RiPP: Lanthipeptide	80%
**17**	NRPS, T2PKS	fredericamycin A	Polyketide: Type II polyketide	96%
**18**	NRPS, LAP	surugamide A/surugamide D	NRP	95%
**19**	NRPS	dudomycin A	NRP	17%
**20**	NI-siderophore	desferrioxamin B	Other	100%

## Data Availability

This genome sequencing project of *S. albidoflavus* MGMM6 has been deposited in the NCBI genome database under NCBI Reference Sequence: CP128384.1. The BioProject and BioSample accessions are PRJNA224116 and SAMN34378109, respectively.
